# Tactical emergency medicine: lessons from Paris marauding terrorist attack

**DOI:** 10.1186/s13054-016-1202-z

**Published:** 2016-02-13

**Authors:** Service Médical du RAID

**Affiliations:** Force d’Intervention de la Police Nationale, Route de Gisy, 91570 Bièvres, France

On the evening of 13 November 2015, three terrorists equipped with military-grade firearms and explosive jackets penetrated Bataclan, a Paris music hall containing 1500 people [1]. The terrorists started a massive execution of the people located in the orchestra pit. Terrorists then moved to the upper circle to take 400 people hostage.

Immediately, RAID (Research, Assistance, Intervention, Deterrence), the French national police counter-terrorism team, and the BRI (Research and Intervention Brigade) were engaged. As previously reported [2, 3], these units encompass police operators and tactical emergency medical physicians, the latter being in charge of rescue planning and care delivery in a tactical environment. At the first look, the three tactical physicians identified more than a hundred casualties, including dozens of fatalities. A terrorist then detonated his bomb, killing himself.

First, RAID police officers and tactical physicians proceeded to zoning. Priority was given to police operation and safety [2]. They delimited a danger zone, referred to as an “exclusion zone”, of which access was strictly limited to RAID/BRI operators because of an explosives threat. Conventional prehospital medical rescue teams remained located outside this police exclusion zone.

While police operators were getting into position within the theater and thus repelling terrorists, two RAID tactical physicians performed triage in the combat zone [2, 4]. They identified about 100 fatalities. Most of the living casualties were identified as invalid. This tactical triage did not aim at identifying relative or absolute emergencies but rather at organizing immediate transfer of the non-invalid patients by themselves to a safe place. A dressing station was located in the theater entrance, far from firearms but still under the threat of explosives. This precluded any conventional rescue team support.

In the combat zone, RAID tactical physicians applied tourniquets to 15 invalid patients [5]. A further 15 patients underwent wound compression with hemostatic dressings. Two received subcutaneous morphine and two received tranexamic acid, and two thoracic exsufflations were performed.

Once salvage therapies were performed [6], more than 50 invalid casualties were moved on physicians’ and operators’ backs from the combat zone to the dressing station; top priority was given to the most severe ones. The purpose of moving these invalid patients was first to secure them in a less dangerous zone, far away from shooting and at a distance from explosives, and to bring them closer to the conventional rescue teams to speed up their subsequent extraction and transfer to a level 1 trauma center [7, 8].

Two tactical physicians joined the operation theater. They perform damage control resuscitation to casualties within the dressing station [9]. Several police officers joined the dressing station and ferried the wounded to the casualty receiving station, where conventional rescue teams were standing under tactical physician supervision. Owing to a stretcher shortage, casualties were first carried out by using crowd barriers.

The initial tactical physicians then joined the assault team to act as forward medical officers. The remaining two terrorists committed suicide, detonating their bombs during a final assault, without causing additional fatalities.

Because the environment remained under the threat of explosives, despite terrorists’ neutralization, conventional rescue teams were maintained out of the exclusion zone until the end of mine-clearing operations. All five physicians continued to perform damage control resuscitation on invalid patients until they were transferred to the casualty receiving station. When conventional rescue teams received clearance to penetrate the theater, all living casualties had already been extracted. The teams discovered 89 fatalities.

Owing to the mismatch between the number of casualties and tactical physicians, the latter first decided to secure non-invalid casualties before treating invalid patients with severe but accessible lesions. The tangle of numerous dead bodies might have hidden living casualties from tactical physicians’ view, preventing the former from receiving care in time.

Not all the concepts of damage control resuscitation were performed. Some of the casualties did not even receive damage control resuscitation, owing first to the aforementioned mismatch between the number of casualties and tactical physicians and second to an insufficient amount of available medical equipment.

Maintaining conventional rescue teams out of danger was consistent with prehospital disaster plans. This appeared a safe option because mine-clearing operators discovered a bag filled with explosives. Of note, despite dynamic adaptation of the exclusion zone boundaries to the threat, post hoc analysis revealed that the distance between the dressing station within the theater and the casualty receiving station was excessive.

## Abbreviations

BRI: Research and Intervention Brigade (*Brigade de Recherche et d’Intervention*)
RAID: Research, Assistance, Intervention, Deterrence (*Recherche, Assistance, Intervention, Dissuasion*)
